# Ripples Make Waves: Binding Structured Activity and Plasticity in Hippocampal Networks

**DOI:** 10.1155/2011/960389

**Published:** 2011-09-27

**Authors:** Josef H. L. P. Sadowski, Matthew W. Jones, Jack R. Mellor

**Affiliations:** MRC Centre for Synaptic Plasticity, School of Physiology and Pharmacology, University of Bristol, University Walk, Bristol BS8 1TD, UK

## Abstract

Establishing novel episodic memories and stable spatial representations depends on an exquisitely choreographed, multistage process involving the online encoding and offline consolidation of sensory information, a process that is largely dependent on the hippocampus. Each step is influenced by distinct neural network states that influence the pattern of activation across cellular assemblies. In recent years, the occurrence of hippocampal sharp wave ripple (SWR) oscillations has emerged as a potentially vital network phenomenon mediating the steps between encoding and consolidation, both at a cellular and network level by promoting the rapid replay and reactivation of recent activity patterns. Such events facilitate memory formation by optimising the conditions for synaptic plasticity to occur between contingent neural elements. In this paper, we explore the ways in which SWRs and other network events can bridge the gap between spatiomnemonic processing at cellular/synaptic and network levels in the hippocampus.

## 1. Introduction

The hippocampus is known to play a critical role in acquiring new episodic memories [1]. The cellular and molecular mechanisms believed to underlie these processes are well characterised and are thought to rely on activity-dependent changes in synaptic transmission which persist for extended periods of time and can be modelled experimentally by the induction of long-term potentiation (LTP) or long-term depression (LTD) [2, 3]. The former is expressed as an increase in the mean magnitude of excitatory postsynaptic potential (EPSP) following stimulation whereas the latter results in an attenuated EPSP. Hebbian plasticity mechanisms, which require repeated and persistent stimulation of the postsynaptic target by the presynaptic cell, are thus able to couple neural elements representing stimuli associated in time and space [4]; the experience-dependent modification in the connectivity of specific neural ensembles is thought to represent the formation of a memory trace [5]. The step between experience and memory is dependent on how structured neural activity—sequences of spiking reliably linked to particular events in past or present behaviour—is able to modify the connectivity patterns of neural circuits. 

Once initiated in the recurrent networks of the hippocampus, newly formed memory traces are fragile and vulnerable to decay and interference [6]. Hence, it is assumed that, following encoding, a process of consolidation must take place in order to stabilise the memory for long-term storage. Neuropsychological data suggest that during consolidation, memory traces become distributed across the hippocampus and connected to areas of the neocortex [7]. The distribution of connectivity patterns across these regions is not well understood, but it is proposed that a transient oscillatory coupling between interconnected neural ensembles is required [8]. 

In recent years, hippocampal sharp wave ripples (SWRs) have been implicated in the process of memory consolidation [9–11]. Ripples are commonly thought to originate in the CA3 region of the hippocampus and propagate via the Schaffer Collaterals (SC) to CA1 and beyond into entorhinal cortex (EC) [12]. However, recent work in transgenic mice has shown that ripples can be observed in CA1 in the absence of SC input, thus some ripples may be driven by input from layer III of the EC via the temporoammonic (TA) pathway or generated intrinsically in CA1 [13]. Described by some as the most synchronous network event in the brain, SWRs entrain large populations of CA3/CA1 neurons into transient but very high frequency oscillations, around 200 Hz in rats but significantly lower in the SC knock-out mice [13, 14]. As a consequence of these high frequency oscillations, near coincident spiking activity between individual elements of the recurrent CA3 network or collateral CA3-CA1 connections occurs on a compressed timescale during the ripple envelope [15]. Such accelerated patterns of correlated activity could provide the optimum conditions for NMDA receptor-dependent plasticity to take place.

Investigations into spike-timing-dependent plasticity (STDP) have revealed the importance of the timing and organisation of pre- and postsynaptic spiking in determining the direction and magnitude of synaptic plasticity. Inducing reliable STDP has been shown to require bursting of pre- and postsynaptic events within a narrow window of opportunity [16–19]—conditions that can be met most efficiently during SWRs. Hence, ripples may be a vital component of the consolidation process by promoting synaptic plasticity within cell assemblies involved in structured neural activity.

Several other factors are also known to have direct impact upon plasticity outcomes within neural networks, including the presence of neuromodulators. The local release of specific neuromodulators such as dopamine, acetylcholine, serotonin, and norepinepherine during different brain states may have a powerful influence over the electrophysiological properties of cells within specific neural networks. For example, in the hippocampus, the neurotransmitter acetylcholine is known to be released during exploratory behaviour and REM sleep, having dramatic effects on the firing properties of pyramidal cells in CA3 and CA1 [20, 21]. This in turn may alter correlated spike activity and therefore the induction of synaptic plasticity. Hence, it is likely that the neural network mechanisms of memory consolidation are constrained by the plasticity potential of activity patterns occurring within a range of oscillatory envelopes and brain states [22].

The hippocampus is likely to remain a key battleground for conflicting theories of spatial and mnemonic processing. However, as we begin to disentangle the most complex and dynamic aspects of hippocampal physiology, we may begin to appreciate the way in which this region could support multiple cognitive functions with considerable mechanistic overlap at the cellular and network level. This paper aims to explore how our developing understanding of hippocampal network phenomena such as SWRs and their impact upon synaptic plasticity could provide a theoretical framework to predict links between neural activity associated with spatial processing and the consolidation of recently acquired episodic information. 

## 2. Online Encoding of Spatial Information

**Figure 1:**
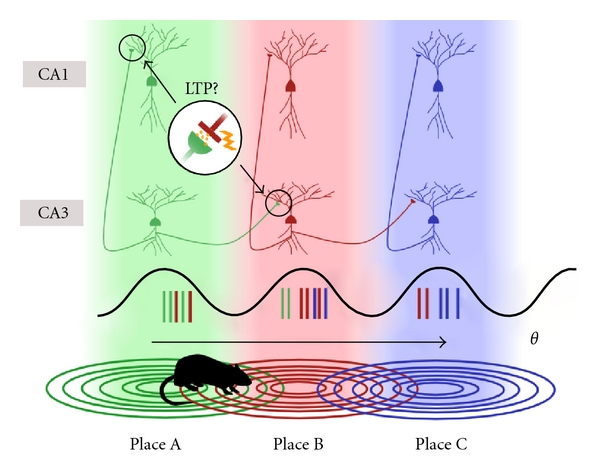
Proposed means of online encoding of spatial trajectories in the rat hippocampus. Place cells in CA3 and CA1 exhibit partial or entire place field overlap. During exploration, hippocampal networks undergo strong 4–7 Hz theta modulation. Overlapping CA3 place cells fire sequentially within the same theta cycle, establishing the conditions necessary for synaptic plasticity to occur between CA3 cell assemblies associated in time and space. Synaptic plasticity could also take place between CA3 and CA1 place cells with the same receptive field as both would fire near simultaneously and are anatomically coupled via the Schaffer collateral to CA1 pathway.

Rodents provide a reliable and widely applicable model for learning and memory that has played an important role in determining the functions of the hippocampus. The location-specific firing of CA3 and CA1 pyramidal (place) cells provides a means of relating online behaviour to the activity of discrete neural assemblies [23–25]. During exploratory behaviour, Cartesian paths can be mapped onto the sequential firing of a series of place cell units with overlapping place fields [26–28] (Figure 1). The dominant model of spatial memory in the hippocampus suggests that the firing of spatially (and therefore temporally) proximal place cells facilitates the online encoding of spatial and episodic memory by promoting plasticity between sequentially active units and ultimately the formation of a memory trace [29, 30]. Over time, individual traces can be integrated into a cohesive, abstract representation of the spatial environment. In conjunction with other location- and orientation-tuned assemblies in entorhinal cortex, spatial maps within the hippocampus could be used for a raft of navigational functions [31]. 

Epochs during which exploration of a spatial environment takes place are dominated by a robust theta frequency modulation of network activity in the rodent hippocampus [32, 33]. The firing of place cells with respect to the phase of the theta cycle changes as the animal moves through a place field, thus theta phase precession provides a means of encoding spatial location independent of firing rate [34]. The degree of spatial overlap between place fields means in any given location the sequential firing of multiple place cells can be compressed into a single theta cycle and their relative order maintained [35]. The coupling of place cell firing across time and space by theta modulation therefore provides an opportunity for synaptic plasticity to take place during periods of online exploration. 

So at which particular synaptic sites might this plasticity take place? The connectivity patterns of the hippocampus suggest that the only region where coactive place cells may be directly synaptically coupled may be the densely recurrent networks of CA3; though the true connectivity ratio of this region is not known, some estimates place it around 1 : 10 [36–38]. CA1 pyramidal cells make few direct axodendritic synapses with other CA1 pyramidal cells [39] but do receive robust innervation from CA3 via the SC, from EC via the TA pathway, and from the CA2 region. An attractive scheme would be one in which replicate traces could be transposed to multiple regions after first being established in the autoassociative networks of CA3. Though direct monosynaptic connections exist between CA3-CA1 as well as CA1-EC and EC-CA3, it seems unlikely that plasticity taking place at these sites contributes to the encoding of spatial or episodic information in the same way as autoassociative connections in CA3. Nevertheless, plasticity at these sites could be contributing to complementary aspects of spatial and episodic encoding and supporting the rapid acquisition role of CA3 [40].

One model, which emphasises the role of local network modifications within CA3 during spatial encoding, is cognitive graph theory [41]. Graph theory suggests that during exploration potentiated pathways between sequentially activated place cells are established. When navigating from one place to another, CA3 is simply searched for the path of least resistance between two place nodes to provide the most efficient route. Despite being perhaps the most widely held working model for CA3 function within spatial processing, cognitive graph theory has little empirical support at a cellular and synaptic level [41, 42]. Indeed, the suggestion that synapses coupling CA3 cells with overlapping place fields can be potentiated during waking exploration has yet to be tested. Tentative support for graph theory can be drawn from work demonstrating that activity recorded from pairs of CA1 place cells or CA3 and CA1 place cells with overlapping place fields is capable of inducing LTP at Schaffer colateral-CA1 synapses *in vitro* [43]. Interestingly, LTP was induced only when the cholinergic agonist carbachol was present in the bath solution, thus mimicking the cholinergic tone present during waking behaviour.

## 3. Offline Consolidation and Trace Stabilisation

**Figure 2:**
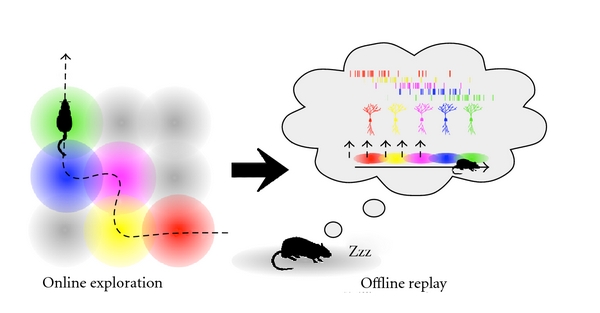
Patterns of activity reflecting spatial exploration during the online state are recapitulated during offline epochs including quiet waking immediately following activity, REM sleep, and slow wave sleep. The greatest degree of temporal overlap in firing patterns occurs between cells with overlapping place fields.

The consequences of online neural activity may only be of transitory importance before a secondary process of consolidation and trace stabilisation takes place during offline epochs that are rest and sleep [5]. The mechanism potentially driving this process at a network level is the reactivation of firing patterns observed in recent behavioural episodes. This reactivation can be considered a “replay” if the pattern of activation occurs in the same temporal sequence as observed during behaviour (see Box 1 of [44] for discussion). The remote replay and reactivation of such waking firing patterns during sleep is a reliably observed phenomenon [45–47] (Figure 2). Replay during sleep has been observed during both REM and non-REM sleep [48, 49], as well as during periods of waking immobility [50, 51] (Table 1). In both cases, waking sequences are replayed at a much faster rate than that observed in the online state, and often in reverse order. Though these examples of replay occur in the hippocampus, the phenomenon has been observed in several brain regions including the ventral striatum and prefrontal cortex [52, 53]. It is not yet clear whether these instances of replay reflect a broader network of structures involved in mnemonic processing or downstream epiphenomena, though hippocampal replay appears to lead replay elsewhere [52, 54].

It is thought that such sleep-mediated reactivation is a vital step in the memory consolidation process [55, 56]. A key sleep state as far as spatial and episodic memory consolidation is concerned is deep non-REM, slow wave sleep (SWS). Specific olfactory or auditory cues paired with visual stimuli during learning can selectively strengthen memory for those stimuli if presented during subsequent slow wave sleep [57, 58]. The presentations of the olfactory cues lead to increased hippocampal activation during the sleep epochs, suggesting that the cues could promote reactivation of newly acquired memory traces, indicating a link between trace reactivation and memory consolidation during SWS. More importantly, this period of sleep also sees the highest concentration of hippocampal SWRs [59], with overall ripple density linked to learning experience [11, 60]. Together this suggests that ripple-related reactivation might have an active role in coordinating the process of offline consolidation.

Both forward and reverse replays are observed during periods of waking immobility following exploratory behaviour [50, 51]; it is possible that these events may fulfil a similar function to replay during SWS in assisting consolidation. However, taken as occurring during online epochs, these instances of replay could reflect other aspects of information processing such as strengthening behaviour-reward associations or decision-making. As it stands, functional differences between sleeping and waking replay remain to be established. 

Spike sequences during ripples occur on a timescale approximately 20 times faster than the rate at which animals move through a sequence of place fields [61]; similar compressed timescale firing sequences occur during locomotion as a consequence of phase precession [35]. Spiking over these timescales would place sequential pre- and postsynaptic activity within the STDP window for LTP, establishing the conditions necessary for plasticity to take place and memory traces to be strengthened or transferred. Despite meeting the temporal requirement for STDP, replay during SWS may have some notable disadvantages for inducing plasticity, the most relevant being the reduced level of cholinergic tone [62]. Cholinergic innervation of CA1 and CA3 is achieved through the release of acetylcholine from septohippocampal cholinergic neuron terminals. Acetylcholine activates multiple cholinergic receptors including muscarinic receptors that serve to facilitate NMDA receptor opening during synaptic transmission via the inhibition of calcium-activated potassium channels [63, 64]. The net effect is to prolong and increase Ca^2+^ transients in the postsynaptic cells and promote the induction of synaptic plasticity. Without the presence of cholinergic tone, place cell activity recorded during the waking state fails to induce plasticity [43]. The question remains whether the degree of temporal compression during ripple-associated replay is sufficient to compensate for the attenuated levels of cholinergic tone and induce plasticity. Alternatively, ripple-associated replay may fail to drive plasticity in the hippocampus in the absence of cholinergic innervation but may still drive plasticity in coupled neocortical regions. In addition the occurrence of eSWRs during exploratory behaviour in the presence of elevated cholinergic tone may permit online plasticity and, in some cases, negate the need for further offline consolidation [65].

The low cholinergic tone present in SWS may also be a vital requirement for SWR generation. Though the precise mechanism through which SWRs are triggered is unclear, it is proposed that their generation in CA3 is dependent on the release of cholinergic suppression of excitatory feedback synapses on recurrent CA3 connections, thus allowing the synchronous depolarisation of the pyramidal cell population in the region [66]. This idea is supported experimentally by the finding that deafferentation of the hippocampus *in vivo* leading to the complete loss of cholinergic input results in enhanced SWR occurrence [67–70]. Furthermore, the occurrence of SWRs has also been observed in hippocampal slice preparations that necessarily lack substantial cholinergic input [71, 72].

Though levels of acetylcholine are strongly linked to current behavioural and therefore neural network states, it does not act alone in modulating the activity of the hippocampus. Other neuromodulators such as serotonin, norepinepherine, and dopamine may all have important roles in regulating plasticity processes involved in spatial and episodic encoding/consolidation. Though their respective effects on membrane properties and synaptic transmission *in vitro *have been explored [73–76], their effects on hippocampal plasticity are less well known. However, it has been shown that the facilitation of LTP *in vivo *by environmental novelty is dependent on the activation of D1/D5 receptors [77], suggesting that dopamine may play an important mechanistic role in the encoding phase at least. As to serotonin and norepinepherine, both have been linked to stress-memory interactions and could serve to modulate encoding and consolidation processes as a result of mood or arousal [78].

## 4. Online Trace Reactivation

Following the identification of SWR events as a key player in offline consolidation processes, the occurrence of hippocampal SWRs during waking epochs is now under increased scrutiny. The so-called eSWRs are known to occur at brief pauses in locomotion as an animal navigates between points in an arena [65, 81]. Their occurrence in such locations has been correlated with both the reactivation of place cell assemblies coding for previously visited locations and subsequent spatial memory performance [82]. These data are some of the first to link reactivation and SWR occurrence with improved behavioural performance, complementing studies that demonstrate impaired performance following ripple disruption during slow wave sleep [9, 10]. Though eSWRs can be regarded as occurring during offline epochs while the animal pauses, their presence in the active waking state serves to blur the boundaries between online and offline processing. However, it should be stressed that eSWRs have so far been linked to reactivation of specific neural assemblies but not necessarily *replay *of extended sequences. By contrast, reverse replay and forward preplay do occur during SWRs during extended periods of immobility following linear track running [50, 51]. Hence, slightly longer pauses during track runs could reveal replay of recent place cell activity during eSWR envelopes. 

Rather than promoting the consolidation of memory traces representing discreet behavioural episodes, it has been suggested that eSWR could be facilitating the spatial processing functions of the hippocampus by helping to establish (and remap existing) place cell assemblies [82, 83]. The reactivation of neural assemblies previously active at other spatial locations may serve to bind novel and existing spatial features within a cohesive place representation. Interestingly, there seems to be a clear dissociation in the functions of CA3 and CA1 to this end; whereas reactivation and reorganisation of place cell assemblies has been observed in CA1, the same has not been observed in CA3 [84]. One suggestion is that CA1 assemblies are able to remap and reorganise rapidly to incorporate new spatial and object information while CA3 provides a more stable representation of the spatial environment.

## 5. Common Network Mechanisms for Spatial and Episodic Encoding

Links between the proposed functional roles of ripple-associated replay in episodic consolidation and spatial processing remain murky and ill defined. Episodic encoding demands the binding of complex spatial and goal-oriented information with a temporal framework. Spatial processing involves establishing a stable internal representation of the environment by integrating information across multiple behavioural episodes; this internal representation may subsequently be used to contextualize memories for single episodes. Episodic and spatial processes may be facilitated independently by synaptic plasticity driven by structured network activity. Nonetheless, it is clear that a substantial degree of mechanistic overlap exists between the two processes at a fundamental level. In both instances, ripple-associated replay and reactivation establish conditions that are favourable for plasticity to take place between neural elements associated in time and space. So intertwined are the spatial and episodic functions of the hippocampus that dissociating their respective mechanisms at a neural network level may be highly challenging. Indeed, it may be almost impossible to separate the encoding of what an animal saw and did at a particular location. The current *in vivo* data do however give us an insight into the mechanistic tools that are in play within hippocampal networks during on/offline consolidation epochs. It is therefore essential that we assess how these activity patterns, present during different epochs and oscillatory envelopes, influence plasticity process at the cellular and synaptic level. Drawing meaningful conclusions from such experiments requires us to frame such findings in the context of a realistic working model for hippocampal network function. 

Theoretical interpretations of hippocampal network functions are manifold [29, 85–88], and it is beyond the scope of this paper to propose yet another. However, it is worth demonstrating how emerging evidence on ripple-related replay could promote plasticity processes within functionally defined sites and states within the hippocampus. Cognitive graph theory makes explicit the need for Cartesian paths to be initially encoded within the recurrent networks of CA3 during online epochs. This is supported by the finding that selective CA3 NMDA receptor knock-out mice which lack plasticity at recurrent CA3 synapses show impaired Morris Water Maze performance and CA1 place cell spatial tuning under spatially novel conditions [40, 89, 90]. Assuming encoding requires an elevation in the synchronicity of firing across distinct neural ensembles in CA3, consolidation could require such a pattern of activity to be transposed to other regions of the hippocampus and wider cortex within a relatively short space of time. 

During offline epochs, the occurrence of SWRs initiates a widespread, high-frequency oscillation of the CA3 and CA1 networks. The traces established during the online encoding period in CA3 are thought to organise activity within discreet neural assemblies in CA1 [91]. It is proposed that only those assemblies that have been organised during the online encoding process are able to fire with the degree of coherence required to induce further synaptic plasticity between their component elements. Likewise noncontingent neural firing producing longer pre- to postsynaptic latencies may lead to LTD between nonassociated elements, thereby increasing the overall signal to noise ratio between the trace to be consolidated and general network activity. This process is likely to lead to both the short-term *in situ* strengthening of the memory trace in CA3 and the possible propagation of organised firing patterns to distinct anatomically coupled neural networks in CA1. Thus, the expression of structured neural activity across CA1 place cell assemblies may arise through direct synaptic connections from the “roots” of the memory trace in CA3. Following such a process, synaptically coupled CA3 and CA1 cells would begin to exhibit a degree of spiking overlap within the same place field. Consequently, this connection could represent two aspects of hippocampal function in both remapping and consolidation. To this end, the Schaffer collateral pathway may be responsible for updating spatial representation as well as transferring behaviourally associated activity patterns during ripple-related replay [92].

Though the CA1 is, in effect, acting as a readout layer for CA3, its rich connectivity patterns with cortical regions would enable it to act as a relay station during memory consolidation [93]. CA1's capacity to connect hippocampal outputs with neocortical structures [94] suggests that neural activity in this region may play a key role in driving extrahippocampal plasticity processes, with the subiculum acting as a relay station capable of modulating downstream projections [95, 96]. The coupling of hippocampo-cortical oscillations has been observed during some behaviours [97, 98], raising the possibly that rhythmic hippocampocortical interactions could be a means of transposing activity patterns from one structure to another with functional consequences. Though the latter study looks at the coherence of theta rhythmic activity, the occurrence of SWRs in the hippocampus has been correlated with increased sleep spindle activity [99, 100]. It has also been demonstrated that activity patterns recoded *in vivo* during sleep spindles are capable of inducing synaptic plasticity in cortical slice preparations [101]; thus, ripples and spindles may be interacting functionally to drive extrahippocampal consolidation processes. Communication between the hippocampus and cortex may also be driven in the opposite direction, with neuronal burst in deep cortical layers triggering discharges relating to SWRs in the hippocampus [102].

## 6. Preplay: Mnemonic Planning or Network Priming?

Many network models of hippocampal function assume that the neural assemblies are naive and receptive to the encoding of novel spatial and episodic memories. However, in reality hippocampal networks are far from naive and are constantly employed in the representation and encoding of numerous streams of information. Emerging evidence raises the possibility that encoding and consolidation may take place following the priming of a recipient neural assembly. The recent discovery of “preplay” activity, where specific sequences of place cell firing patterns seen during a spatial task are shown to be active in the same sequence immediately prior to the behaviour, suggests this may be the case [103]. This striking finding can be rationalised in a number of ways; either an animal constructs forward plans relating to future behaviour, or the hippocampus is able to identify neural assemblies which are available to encode new spatial information. Though the latter seems far more plausible, it is unclear how this process takes place and whether this could represent a mechanism used to search for and prime free capacity within the hippocampal network. At a synaptic level, such activity could serve to bias plasticity mechanisms within specific cell assemblies, making them more receptive to changing patterns of activation. Alternatively such patterns could emerge merely as a consequence of prior plasticity.

## 7. Reconsolidation and Remapping

So far, we have considered how hippocampal circuitry, network oscillations, and structured neural activity could take part in an integrated scheme of spatial/episodic memory encoding and consolidation. In isolation, stored engrams represent little more than patterns of potentiated connectivity between associated neural stimulus representations. Not all of these engrams will be entirely novel, and many episodes will possess a large degree of featural overlap with existing memories. Therefore, the process of updating and reconsolidating previously stored information is bound inextricably with the process of creating new memories and spatial representations. In this sense, replay and reactivation of waking activity patterns may be as important in loosening existing representation as firming up novel ones. In this section, we discuss how replay and reactivation could facilitate the updating of existing episodic memories and the reconfiguration of place cell mappings in the hippocampus. 

Mounting evidence from humans support the prevailing hypothesis that updating existing memories requires the reactivation and transient destabilization of previously stored memories [104–106]. During this period of “reconsolidation”, memories once again become vulnerable and liable to interference [107, 108]. However, the risk of potential interference and degradation is much lower if memories are reactivated during SWS than wakefulness as both the level of external input and cholinergic tone in hippocampal networks are lower [22]. Replay during sleep could promote both the safe reactivation of existing hippocampus-dependent memories and the effective incorporation of newly acquired information. While reconsolidation is taking place, hippocampal-cortical connectivity is reestablished [109], yet it is unclear whether the coupling of the two networks represents the retrograde flow of existing information back to the hippocampus where it can be integrated with novel traces, or whether it represents the downstream flow of updated patterns to the cortex. The pattern of excitation produced by SWRs during SWS suggests that the latter may be the more likely scenario.

Whilst the destabilisation and interference caused by waking reactivation may be undesirable in the context of long-term autobiographical memory consolidation, the opposite may be the case for spatial navigation [110]. Precise navigational capabilities may depend upon the ability to build accurate spatial representation of the environment. Place cells may be a key component of the neural mechanisms set up to facilitate this. Omnidirectional place cell firing characteristics can be established relatively rapidly following entry into a novel spatial environment [23, 27, 111]. However, following a change in the spatial configurations of the environment, place cells may be forced to remap to the new surroundings. In such a scenario, it would be desirable for cell assemblies to be reactivated and primed for reorganisation.

Replay during sleep may provide the ideal opportunity for hippocampal place units to remap and update their spatial representations. During remapping, place cells in CA3 and CA1 representing the same area of Cartesian space must adapt to the new configurations, a process thought to involve LTP within and possibly between cell assemblies in the two regions [112]. However, evidence suggests that CA3 and CA1 remapping occurs sequentially and not simultaneously, with CA3 exhibiting much more rapid spatial reorganisation [93, 113]. The transferral of accurate, stable, and updated spatial representation from CA3 place cells to their CA1 counterparts is a process that is likely to involve plasticity at Schaffer collateral synapses. This process could be driven by the propagation of activity from CA3 to CA1 during SWRs (or waking eSWRs), though it remains uncertain whether the different stages of the remapping procedure take place during on or offline epochs. Novel lines of investigation could involve analysing plasticity processes induced by patterns of CA3 and CA1 cell activity recorded before, during, and after spatial remapping or the effect of NMDA receptor antagonists on CA1 place field stability.

## 8. The Role of REM

With such a large proportion of the network level investigation of hippocampal function currently focused on the role of SWS and SWRs, the part played by REM sleep is often neglected. REM is characterised by strong hippocampal theta power and relatively high levels of cholinergic tone. Though extended periods of remote hippocampal replay have been observed during REM sleep [48], the temporal compression of firing sequences is much less than that seen during SWS, only 1.5 times faster than that seen in waking sequences compared with 20-fold increase in SWS. However, there is little evidence to suggest that REM sleep plays an active role in the consolidation of long-term episodic or spatial memories [114]. No immediate deficit in declarative memory performance has been observed following REM sleep deprivation, but experience-dependent activity following procedural learning has been observed during REM sleep [55], suggesting that REM replay may have a complementary role to SWS in memory consolidation. REM sleep in humans also coincides with episodes of dreaming, when autobiographical, emotional [115], and procedural elements of past experience can be recalled in an unstructured and abstract fashion. The occurrence of dreaming and procedural consolidation in REM sleep has led to the suggestion that REM reactivation may be involved in the processing and integration of memory traces, enabling them to be recalled fluently and used flexibly during conscious thought and activity [108, 116–119]. Such a process is unlikely to require the encoding of extended behavioural sequences but could utilise plasticity processes in coupling sequences coactivated during dream states and strengthen feature overlap between related episodes.

## 9. Disrupted Sleep and Memory Processing in Disease

As a clear picture of the network mechanisms of memory formation emerges, it is important to ask how they may be disrupted in the disease states. It is clear that sleep has a vital role in memory consolidation by establishing the appropriate brain states for secure replay and reactivation to take place. Hence, the very structure of sleep itself determines how information is processed in the offline state. Sleep disorders and sleep disruption are common symptoms seen in a wide range of neurological and psychiatric conditions such as schizophrenia [120]. Changes in the structure of sleep and particularly reduced slow wave sleep and spindle activity are reliably observed in schizophrenic patients [121, 122], such deficits may contribute to the cognitive impairment seen in some individuals. Whether this relates to or results in altered synaptic plasticity in these diseases remains to be established.

## 10. Conclusions

Activity within hippocampal networks is highly dynamic, yet these networks support the storage of stable and accurate representations of our environment and experiences. Driving these changing patterns of plasticity are both externally generated behavioural inputs and internally generated network oscillations. The ongoing interaction between these two factors results in the pattern of activity that can be observed *in vivo *as structured firing of pyramidal cell populations. The plasticity processes induced as a result provide the basis of learning and memory. 

The transition between structured activity and lasting memory trace remains poorly understood, but detailed observation of the key cellular and network phenomena provides an insight into the underlying mechanisms of episodic memory and spatial processing. What was considered to be a two-stage process involving clear distinctions between encoding and consolidation is now known to be much more dynamic and multilayered. The evidence reviewed above suggests a scheme in which online exploratory activity leads to rapid changes in the patterns stored in the recurrent networks of CA3 though the induction of synaptic plasticity between place cells. High cholinergic tone during phases of exploration ensures that plasticity is reliably induced. Pauses in exploration allow eSWR to occur and permit the rapid reactivation and remapping of CA1 place cell assemblies. During extended periods of rest and inactivity, recent patterns of place cell activity are replayed in reverse order, strengthening newly potentiated pathways between overlapping CA3 place cells. Subsequent slow wave sleep allows patterns of connectivity established in CA3 to be transferred to CA1 and neocortex during SWR-associated replay events. Replay within high-frequency oscillatory envelopes overcomes the inhibitory effect on synaptic plasticity imposed by low levels of cholinergic tone. Intervening REM epochs allow newly consolidated information to be bound within existing mnemonic representations and permit further consolidation in conditions of elevated cholinergic innervations. 

Though the conceptual framework outlined above is tentative and does not capture all of the available evidence, it highlights the key role played by SWR in binding the multiple aspects of hippocampal physiology involved in the encoding and consolidation of spatial and episodic memory.

## Figures and Tables

**Table 1:** Studies showing the reactivation and replay of waking activity patterns in the hippocampus during a range of behavioural and brain states.

Paper	Activity pattern	Brain state	Predicted levels of acetylcholine	Network oscillations	Behavioural paradigm
Wilson and McNaughton [46]	Place cell reactivation	SWS	Low	SWRs	Four arm tracks
Skaggs and McNaughton [45]	Sequential place cell reactivation	SWS	Low	SWRs	Triangular track
N$\overset{´}{\text{a}}$dasdy et al. [61]	Place cell replay	SWS	Low	SWRs	Wheel running
Louie and Wilson [48]	Remote forward place cell replay	REM	High	Theta	Circular track
Lee and Wilson [49]	Remote forward place cell replay	SWS	Low	SWRs	Linear track
Foster and Wilson [50]	Local reverse place cell replay	Awake	High	SWRs	Linear track
Diba and Buzsáki [51]	Local forward and reverse place cell replay	Awake	High	SWRs	Linear track
Csicsvari et al. [79]	Local reverse place cell replay but reduced remote replay	Awake	High	SWRs	Exploration in rectangular box.
Karlsson and Frank [80]	Local and remote forward replay	Awake	High	SWRs	“E”-shaped maze
